# Smart Homes for Elderly Healthcare—Recent Advances and Research Challenges

**DOI:** 10.3390/s17112496

**Published:** 2017-10-31

**Authors:** Sumit Majumder, Emad Aghayi, Moein Noferesti, Hamidreza Memarzadeh-Tehran, Tapas Mondal, Zhibo Pang, M. Jamal Deen

**Affiliations:** 1 Department of Electrical and Computer Engineering, McMaster University, Hamilton, ON L8S 4L8, Canada; majums3@mcmaster.ca; 2 Department of Network Science and Technology, Faculty of New Sciences and Technologies, University of Tehran, Tehran 141746-6191, Iran; emad.aghayi@ut.ac.ir (E.A.); m.noferesti@ee.kntu.ac.ir (M.N.); hmemar@ut.ac.ir (H.M.-T.); 3 Department of Pediatrics, McMaster University, Hamilton, ON L8S 4L8, Canada; mondalt@mcmaster.ca; 4 ABB Corporate Research, 721 78 Vasteras, Sweden; pang.zhibo@se.abb.com; 5 School of Biomedical Engineering, McMaster University, Hamilton, ON L8S 4L8, Canada

**Keywords:** smart home, telemedicine, telehealth, health monitoring, aged people, smart care, gerontechnology

## Abstract

Advancements in medical science and technology, medicine and public health coupled with increased consciousness about nutrition and environmental and personal hygiene have paved the way for the dramatic increase in life expectancy globally in the past several decades. However, increased life expectancy has given rise to an increasing aging population, thus jeopardizing the socio-economic structure of many countries in terms of costs associated with elderly healthcare and wellbeing. In order to cope with the growing need for elderly healthcare services, it is essential to develop affordable, unobtrusive and easy-to-use healthcare solutions. Smart homes, which incorporate environmental and wearable medical sensors, actuators, and modern communication and information technologies, can enable continuous and remote monitoring of elderly health and wellbeing at a low cost. Smart homes may allow the elderly to stay in their comfortable home environments instead of expensive and limited healthcare facilities. Healthcare personnel can also keep track of the overall health condition of the elderly in real-time and provide feedback and support from distant facilities. In this paper, we have presented a comprehensive review on the state-of-the-art research and development in smart home based remote healthcare technologies.

## 1. Motivation

In the last few decades, the life expectancy in most countries in the world has increased dramatically. This improvement is achieved primarily due to significant advancements in medical science and diagnostic technology, as well as the rising awareness about personal and environmental hygiene, health, nutrition, and education [1,2,3,4]. However, increased life expectancy coupled with falling birthrates is expected to result in a large aging population in the near future. In fact, according to the World Health Organization (WHO), the elderly population over 65 years of age would outnumber the children under the age of 14 by 2050 [3]. In addition, about 15% of the world’s population suffers from various disabilities, with 110–190 million adults having significant functional difficulties [5]. People with disabilities, due to their limited mobility and independence, are often deprived of their regular healthcare needs. Furthermore, chronic diseases and conditions such as heart disease, stroke, cancer, and diabetes are among the most common health problems in adults. Half of all American adults aged 18 years or older are reported to have at least one chronic condition with one in three adults suffering from multiple chronic conditions. Out of 10 leading causes of death, chronic diseases account for ~65–70% of total mortality [6]. In particular, heart disease and cancer together are the leading causes of death, accounting for 48% of all deaths [7]. In addition, unregulated blood sugar i.e., diabetes, if not managed properly, may lead to long-term complications such as kidney failure, limb amputations, and blindness. 

Therefore, it is no wonder that the demand for healthcare services increases with the increasing average life expectancy of the population. However, the cost associated with present-day healthcare services continues to rise due to the ever-rising prices of prescription drugs, diagnostic tools and in-clinic care. For example, investments in healthcare sectors increased by a massive $11.5 billion in the 2017 budget of Ontario, Canada [8]. Therefore, existing healthcare services are likely to impose a significant burden on the socio-economic structures of most countries, particularly the developing and least developed ones [9,10,11,12,13]. In addition, a large number of elderly people require regular assistance for their daily living and healthcare, which are mostly supported by the family, friends or volunteers [14]. Formal paid care services offered by caregivers, or elderly care centers are expensive and thus are still out of reach for a large section of the elderly population living under constrained or fixed budget conditions [15,16]. Therefore, there has been a growing awareness to develop and implement efficient and cost-effective strategies and systems in order to provide affordable yet superior healthcare and monitoring services for the people having limited access to healthcare facilities, particularly the aging population.

The elderly may require frequent, immediate medical intervention, which may otherwise result into fatal consequences. Such emergency situations can be avoided by monitoring the physiological parameters and activities of the elderly in a continuous fashion [16,17,18]. In most emergency cases, the elderly seek in-patient care, which is very expensive and can be a serious financial burden on the patient if the hospital stay is prolonged. Remote health monitoring in a smart home platform, on the other hand, allows people to remain in their comfortable home environment rather than in expensive and limited nursing homes or hospitals, ensuring maximum independence to the occupants [19]. Such smart homes are outfitted with unobtrusive and non-invasive environmental and physiological sensors and actuators that can facilitate remote monitoring of the home environment (such as temperature, humidity, and smoke in the home) as well as important physiological signs (such as heart rate, body temperature, blood pressure and blood oxygen level), and activities of the occupants. It can also communicate with the remote healthcare facilities and caregivers, thus allowing the healthcare personnel to keep track of the overall physiological condition of the occupants and respond, if necessary, from a distant facility [16,20].

## 2. Introduction

In recent years, the Internet-of-Things (IoT) has gained much attention from researchers, entrepreneurs, and tech giants [21,22,23] around the globe. The IoT is an emerging technology that connects a variety of everyday devices and systems such as sensors, actuators, appliances, computers, and cellular phones, thus leading towards a highly distributed intelligent system capable of communicating with other devices and human beings [21,22,23]. The dramatic advancements in computing and communication technologies coupled with modern low-power, low-cost sensors, actuators and electronic components have unlocked the door of ample opportunities for the IoT applications. Smart home with integrated e-health and assisted living technology is an example of an IoT application in gerontechnology that can potentially play a pivotal role in revolutionizing the healthcare system for the elderly. As the world is rapidly moving towards the new era of the IoT, a fully functional smart home is closer to reality than ever before.

**Figure 1:**
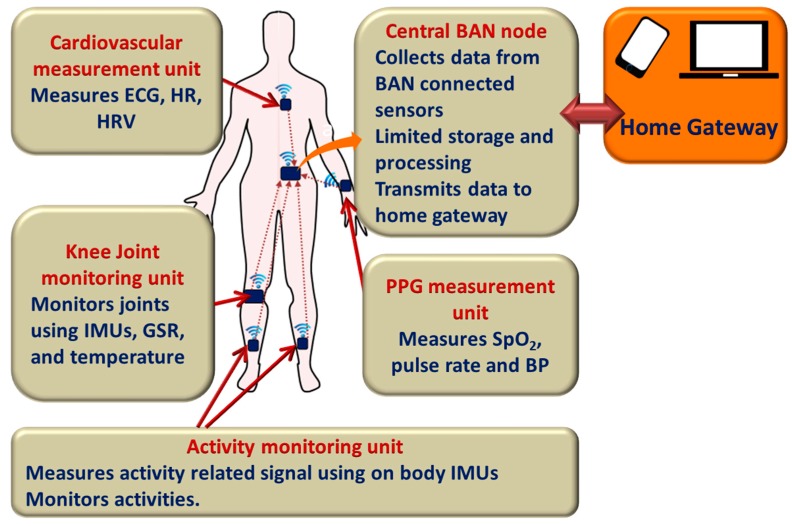
Wireless Body Area Network (WBAN) for wearable medical sensors.

In a smart home, sensors and actuators are connected through a Personal Area Network (PAN) or Wireless Sensor Network (WSN). Wearable biomedical sensors such as electrocardiogram (ECG), electromyogram (EMG), electroencephalogram (EEG), body temperature and oxygen saturation (SpO_2_) sensors can be connected in a Wireless Body Area Network (WBAN) or Body Sensor Network (BSN) in order to obtain automated, continuous, and real-time measurement of physiological signals. The central BSN node collects all physiological data, performs limited data processing and functions as the gateway to the PAN/WSN. The actuators operate based on the feedback from the occupants or from the central computing system. The central computing system collects environmental, physiological and activity data through the PAN/WSN, analyzes them and can send feedback to the user or activate the actuators to control appliances such as humidifier, oxygen generator, oven and air conditioner. It also functions as the central home gateway, which sends measured data to the healthcare personnel/service providers over the internet or the cellular network. In order to realize communication between all wireless sensors and actuators, standard protocols from Wireless Sensor Networks (WSNs) and ad-hoc networks are used. However, current protocols designed for WSNs are not always applicable to WBAN [24,25,26]. An illustration of a medical WBAN used for patient monitoring is shown in Figure 1. Multiple sensors can be placed over clothes or directly on the body, or implanted in tissue, which can facilitate measurement of blood pressure, heart rate, blood glucose, EEG, ECG and respiration rate [27]. Some applications of WBAN from the literature, which are important for smart home, are presented in Table 1. 

The market penetration of smart home has seen a steady rise over the past few years. The revenue of the global smart homes market in 2014 was at $20.38 billion and it is projected to grow to $58.68 billion by 2020 [28]. Unfortunately, the smart home market is currently in a state of stagnation primarily due to the high price of components, limited demands, long replacement periods as well as consumers’ reluctance to adopt currently available complex systems, which require multiple networking devices and software applications to implement and control the smart homes [29]. In addition, ensuring the privacy and security of the sensitive medical and personal information is critical for achieving widespread acceptance among consumers [19]. However, with the continuous advancement of high-speed computing and secured communication technologies coupled with miniaturized low-power and low-cost sensors and actuators, it is expected that the smart home market will flourish dramatically in the coming years, thus leading towards ‘smart cities’ [30].

In this article, we present a review on the current state of research and development in smart homes with a primary focus on remote healthcare services. The concept of remote health monitoring is studied in Section 3, which is followed by a discussion (Section 4) on the architecture and standards of an IoT-based smart home for remote health monitoring. Some recent works on different forms of remote healthcare services are presented in Section 5. In Section 6, we present some key prototypes and commercially available solutions of smart homes. Some key challenges in realizing fully functional smart homes are discussed in Section 7. Finally, the paper is concluded in Section 8 with some future perspectives of smart homes. 

## 3. Remote Health Monitoring

Modern sedentary lifestyles and food habits coupled with large aging population have resulted in a rising tide of chronic diseases such as heart disease, obesity, diabetes and asthma [19]. According to the World Health organization (WHO), cardiovascular diseases are currently responsible for most deaths around the globe [37]. In addition, diabetes is rising dramatically and expected to be the seventh leading cause of death in 2030 [38]. Furthermore, poor outdoor air quality in most industrial and big cities is giving rise to cancer, cardiovascular and respiratory diseases such as asthma, and lung diseases. Around 235 million people are currently suffering from asthma and an estimated 383,000 people died from asthma in 2015 [39]. Although, chronic diseases are among the most common and costly health issues, they can be prevented by early detection through long-term monitoring or controlled effectively through appropriate management, thus allowing people to enjoy a good quality of life [40]. However, shortage of skilled healthcare personnel, limited budget, increasing healthcare cost coupled with growing healthcare needs [41] are the critical constraints for long-term monitoring and management of health. Therefore, an affordable, un-obtrusive and comprehensive healthcare solution with minimal workforce is of utmost importance for long-term health management and monitoring, especially for the rapidly rising elderly population. 

### 3.1. E-Health and M-Health

**Figure 2:**
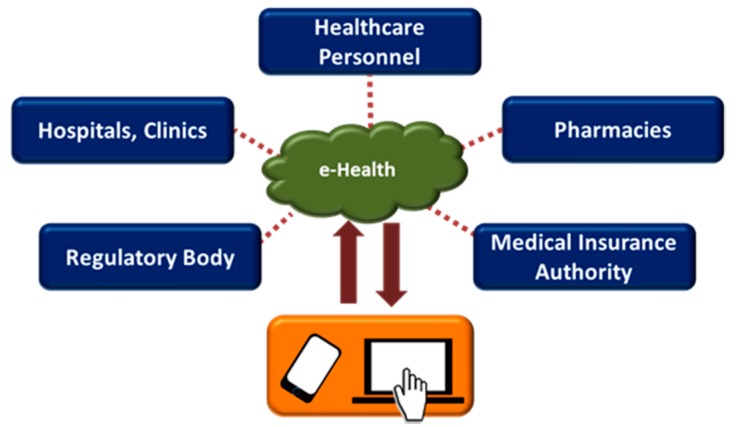
E-Health Infrastructure.

E-health utilizes information and communication technologies to digitize and automate healthcare processes and tasks, thus enabling services like e-prescription, e-supply and e-records [42] for patients. For example, electronic medical records (EMRs) or electronic health records (EHRs) can store and provide complete and detailed information about the medical history of patients, which can be accessed remotely and used by the authorized healthcare personnel for decision-making [43,44,45]. Modern information and communication technologies allow continuous monitoring and recording of physiological parameters/signals, which can be stored in a central secured database. These records can be made readily available to the authorized personnel such as caregivers, emergency medical services (EMS) and family doctors when needed. A fully functional E-health system may lead towards an efficient, high quality and ubiquitous healthcare service at a lower cost and with minimal error. The infrastructure of E-health is illustrated in Figure 2.

However, the advancement of compact, portable communication and computing devices such as smartphones, and tablets has created the pathways for the evolution of M-health from the classical E-health concept. M-health is based-on modern mobile communication technologies such as Enhanced Data GSM Environment (EDGE), 3G, High Speed Packet Access (HSPA), and Long-Term Evolution (LTE), which offer high-speed and seamless data transfer from anywhere, at any time, thus allowing people to remain connected with the central M-health system.

As mentioned earlier, a network of environmental, biomedical, and motion sensors, which can measure and send measured data to the remote facilities through the gateway, can be deployed at home. Wearable biomedical sensors located in (implanted devices, e.g., pacemaker, insulin injector), on (e.g., ECG or EEG electrodes), or around (e.g., gesture detectors, external devices) the human body can be connected in a BAN, thereby enabling ubiquitous, un-obtrusive continuous health monitoring. The data collected by the sensors are transmitted to a central BAN node, which can process and transfer information to an external device (the user’s mobile phone) or a remote workstation (a nurse station in a hospital or nursing home). Monitoring of key parameters such as patient’s activity, heart rate (HR), blood pressure (BP), respiration rate (RR) and body temperature (BT) from a remote station and sending feedback accordingly over the M-health system may potentially lead towards the E-ambulatory care system [46]. 

It is expected that modern miniature sensing and actuating technologies along with the advanced connectivity platforms such as BAN and home based WSN as well as increased penetration of high-speed internet globally will play a pivotal role in moving towards the home-based remote healthcare services from the conventional in-clinic care. The network of sensors monitors the condition of the subject under supervision and sends the information to a distant healthcare facility over the internet [47] or it can automatically call for EMS in case of an emergency. However, ensuring seamless connectivity, secured transmission channels and data storage are the key challenges in developing a complete infrastructure of an E-health or M-health system for medical information management and continuous monitoring of health. Moreover, interoperability among different protocols and standards is also critical for the consistent operation of the system. In addition, precise and accurate measurements of key health parameters are vital for a reliable health monitoring system. 

### 3.2. Home-Based Remote Health Monitoring

The advancement of miniaturized and inexpensive sensors, embedded computing devices, and wireless networking technologies paved the way for realizing remote health monitoring systems. Remote health monitoring allows un-obtrusive, ubiquitous, and real-time monitoring of physiological signs without interrupting the daily activities of individuals. People can remain in their familiar home environment and enjoy their normal lives with the friends and family while their health is being monitored and analyzed from a remote facility based-on the physiological data collected by different on-body sensors. The system can perform long-term health trend analysis, detect anomalies, and generate alert signals in the case of an emergency. 

In order to facilitate continuous monitoring of health, various E-health devices are proposed in the literature [48,49]. EnViBo, which stands for embedded network for vital sign and biomedical signal monitoring, is such a platform for the ambulatory monitoring of adults with medical conditions or people working under extreme conditions such as firefighters and rescue personnel [50]. An open-source platform for a wireless body sensor network named DexterNet was presented in [49]. This platform comprises a body sensor layer (BSL), a personal network layer (PNL), and a global network layer (GNL) that supports real-time and persistent human monitoring in both indoor and outdoor environments [49]. 

Telemedicine is an advanced form of E-health service which provides remote healthcare support, analyzes the trends in medicine usage and makes the information available to the authorized personnel with the help of modern communication technologies, thus allowing faster and affordable healthcare services [51]. In a recent study [52] on the effectiveness of telemedicine, it was found that telemedicine was beneficial to reduce mortality due to different causes. Telemedicine also effectively reduces hospital admission, length of stay and mortality due to heart failure [52]. A telemedicine system for in-home monitoring of vital signs is presented in [36]. The sensors communicate with an android based smartphone using Bluetooth. The smartphone functions as the gateway to a long range communication network such as a 3G cellular phone network or wireless local area network (WLAN). A tele-medicine system that can measure several physiological signs of the resident and send them to a computing platform for further analysis was developed and reported in [53]. The medical staffs can keep track of the signs over a web-based interface. The system is capable of transmitting real-time information to a remote medical server over both the cellular networks and internet in case of an emergency or on request. Some technology companies are currently offering tele-medicine services over web-based platform [54,55,56,57]. The services include secure video communication between doctor and patients, remote health monitoring, and emergency care service.

Intelligent furniture such as smart chairs and smart beds can also be utilized for measuring physiological data at home. For example, a smart bed can monitor the health status and sleep patterns of an individual. It can also be used to detect a heart attack of the cardiac patients while they are on the bed or sleeping. The system can immediately inform the central system, caregivers, EMS or any authorized personnel in an automatic fashion, thus reducing the risk of fatality.

## 4. Internet-of-Things and Connected Homes

**Figure 3:**
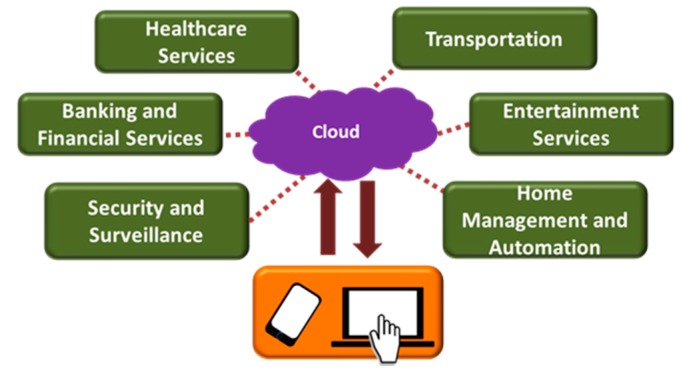
Applications of the Internet of Things (IoT).

The developments of low-power wireless communication technologies, miniaturized sensors and actuators as well as growing penetration of internet, tablets, and smartphones are leading us towards the new era of the IoT [21]. Connected homes or smart homes use the concept of the IoT, which offers a platform to monitor safety and security of the home or to automatically control the home environment or appliances, over the internet from anywhere. The IoT can be defined as a network of intelligent objects that is capable of organizing and sharing information, data and resources, decision making, and responding to feedback [58]. It allows human-to-human, human-to-things and things-to-things interaction by providing a unique identity to each and every object [59]. The US National Intelligence Council (NIC) considered the IoT technology as one of the six disruptive civil technologies that can potentially impact US national power [60]. Some researchers envisioned the IoT as an emerging field that can enable new ways of living by bridging the physical world with the digital computing platform by means of smart sensing and actuating devices, and appropriate communication technologies such as Bluetooth Low Energy (BLE), ZigBee and ANT [61,62,63,64]. Therefore, the concept of IoT can be exploited in a wide range of applications (Figure 3) such as E-health, assisted living, enhanced learning, intelligent transportation, environmental protection, government work, public security, smart homes, intelligent fire control, industrial monitoring and automation [65]. 

Traditional homes, in spite of being energy-hungry, are generally not designed to monitor the environment of the home, or physiological conditions and activities of the occupants by itself [63]. A smart home, in contrast, is a traditional house embedded with smart devices and modern communication technologies that can facilitate remote and automatic monitoring of home environment, security and overall health status of the occupants. However, in order to achieve widespread acceptance among the users, smart homes need to be affordable. Therefore, low-power and efficient communication technologies and public networks, along with low-cost devices are critical for smart homes. In addition, several key technological challenges such as full interoperability among the interconnected devices, high degree of precision and accuracy, processing resource limitation, and privacy and information security need to be addressed [19]. A successful implementation and penetration of fully-fledged smart homes may lead towards smart cities or intelligent residential districts in the near future [65,66]. 

### 4.1. Layered Architechture of Smart Home

**Figure 4:**
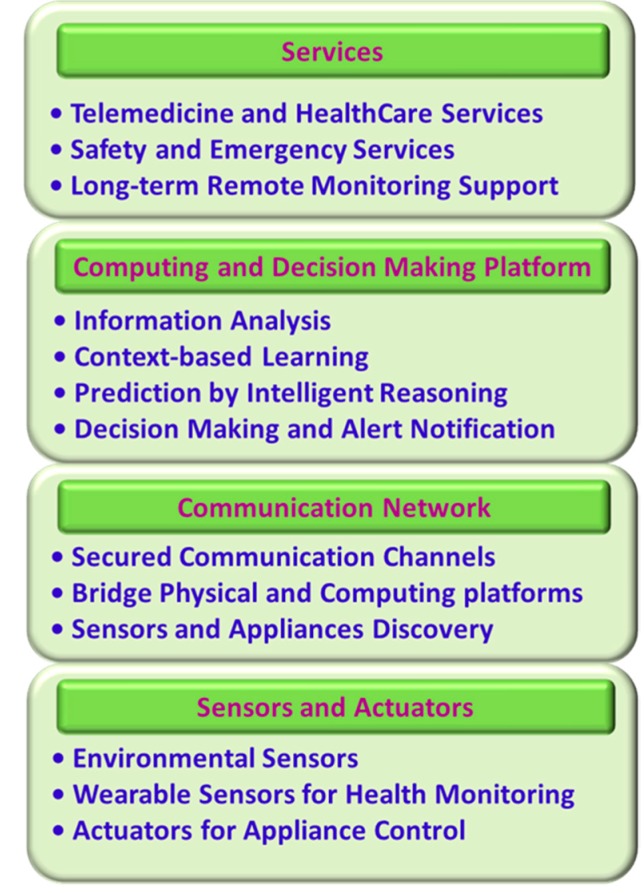
A four layer architecture for smart home.

Smart homes may include a set of environmental, activity and physiological sensors, actuators connected through a wireless communication medium. The advancement in low-power, smaller dimension sensing, actuating and transceiver systems coupled with modern communication technologies and inexpensive computing platforms such as field programmable gate array (FPGA), microcontrollers, microprocessors paved the way for low-cost smart home systems. A four-layer architecture for smart home [18] is presented in Figure 4.

#### 4.1.1. Sensors and Actuators

Sensors and actuators play the key role in smart home by bridging the gap between the physical world and the digital domain. Smart homes use several sensors to collect data about the home environment such as light illumination level, temperature, pressure, gas leakage, oxygen level, and about the activity or location of the occupants by using inertial measurement units, RFID tags [67,68] or passive infrared (PIR) [50,69] sensors. Physiological parameters such as BP, HR, SpO_2_, galvanic skin response (GSR), RR can be measured using wearable sensors. Actuators can respond to the feedback from the occupants or from the central decision making platform by performing small scale maneuver to control environment or to deliver drugs such as insulin on occupant’s body. These sensors and actuators can communicate with the central computing and decision making platform over the wireless communication medium. The sensors, particularly the wearable medical sensors need to be energy efficient and unobtrusive in order to facilitate long-term monitoring. Sensors and actuators with embedded energy harvesting technologies [70] can effectively increase the running time of the ambulatory devices.

#### 4.1.2. Communication Network

All sensors and actuators in the smart home are connected with the central communication and decision making platform though a communication network, which forms the second layer of the smart home architecture. All physiological and environmental signals measured by the sensors are transmitted to the central computing node over a wireless and/or wired communication medium. Although wired connection is a feasible solution for fixed-position based environmental sensors, it is not suitable for wearable and long-term monitoring systems. Wired connections for the wearable BSN may cause inconvenience to the user and restrict users’ mobility. It may also cause occasional connection failure among the on-body sensors. Textile based conductive medium such as conductive fabrics can be used to communicate with the on-body sensors as an alternative to the wired connection [71,72]. Conductive fabrics can be produced using conventional textile technologies such as weaving, stitching, embroidery, and screen printing. However, conductive textiles suffer from low durability and limited washability, thus resulting in poor or failed connectivity after prolonged use [16]. Therefore, modern low-power wireless communication technologies appear to be the most viable and reliable medium for short-range communication. Table 2 presents the key features of some commonly used wireless technologies for short range communication. 

The wearable medical sensors can be connected in a BSN, where the central BSN node is connected with all environmental sensors and actuators through the WSN. All the sensors and actuators in the smart home are connected to form a Local Area Network (LAN) or Personal Area Networks (PAN) and to provide data communication inside the smart home [73]. The central decision making platform can communicate with any sensors and actuators in the network using the WSN to collect data or send feedback to perform necessary actions, if required. 

#### 4.1.3. Computing and Decision Making Platform

The third layer of smart home architecture is responsible for computing and decision making, thus functioning as the brain of the system. This layer is equipped with computing system such as smartphone, computer or custom-built processing node based on Field Programmable Gate Array (FPGA) or microprocessors. It gathers data from the sensors and actuators over the WSN, processes, and analyzes measured data, and sends feedback to the user or to the actuators. It may also store measured data, display the results to the user, and may run prediction algorithms. The prediction algorithms can exploit the features of artificial intelligence (AI) and make use of deep learning [74,75,76,77] and machine learning techniques [78,79,80,81,82] such as artificial neural network (ANN), support vector machine (SVM), and K-Nearest Neighbors (KNN) to learn and develop models for the home environment as well as for the behavioral and physiological patterns of the occupants. Researchers from the University of Missouri, Columbia equipped an independent senior living facility, called TigerPlace [83], with smart sensors to monitor and assess the residents’ activity and overall health [84]. A wide variety of sensors were installed to monitor occupants’ daily activities, pulse and respiration. The researchers, however, initially developed a fuzzy-logic based model that can produce linguistic summaries of two activities—movements in bed and movements in the apartment—by analyzing the motion sensor data collected over a longer period of time. The work may be further extended to incorporate more sensor data and detect anomalies by assessing the magnitude of deviations from the normal patterns in the activities and physiological data. A detailed discussion on different machine learning and data mining techniques used in smart home application was presented in [75,76,77,81,82]. 

Such models are used by the computing platform to make predictive decisions about the home environment or occupant’s health status based on the information received from several sensors. The adoption of AI will also allow this platform to exploit robotics [85,86] to control the smart home peripherals and to provide services to the occupants in an automatic fashion with continuous improvements in accuracy and precision over time. One such platform, Lab-of-Things (LoT) is developed by Microsoft Research that uses an operating system named HomeOS to monitor, manage, and control interconnected devices in homes and analyze data received from the sensors [87].This layer is also responsible for ensuring a secured, long-range communication channel to the remote service provider. It can transmit the measured data, key physiological or environmental parameters over the internet or cellular network, thus functioning as the home gateway to the remote facility. This platform monitors and assesses the measured physiological or environmental data continuously. If any abnormality in the home environment or in the vital signs of the user’s health is detected, it can raise an alarm or send alert messages to the service providers in the form of voice call, text message or e-mail. 

#### 4.1.4. Services

The top layer of the smart home architecture consists of the services delivered to the user by the service providers. These services may be associated with the health of the occupants, environment, safety, or security of the home and the residents. Services provided to the smart home can be tailored according to the requirements of the occupants based on the level of medical attention or safety and security required. In a smart home, the gateway platform functions as the primary service provider, for example, by activating necessary actuators to control the home environment, door locks or dosage, in the case of automated drag delivery. The gateway system may adopt AI technologies to assess the safety, security and environment of the home and control the smart devices to provide the occupants with better services [78,79,80,85,86]. The gateway can learn and keep continuous track of the occupants’ physiological conditions with the help of the BSN-connected wearable health sensors. The AI technologies implemented in the gateway will allow the smart devices in the smart home to be controlled to adjust the home environment according to the occupants’ requirement. It can also monitor the home environment and can detect any hazardous situation such as presence of smoke or gas leakage using the environmental sensors installed at different places in the home. In case of any anomalous physiological or environmental conditions, the gateway raises alarms and sends electronic notifications such as emails, text messages, and phone calls to the secondary service provider. 

The secondary service provider is the central hub of all the subscribed smart homes and responsible for management, maintenance, connectivity, and information security of the smart home network and systems. It continuously monitors for alarms or emergencies and immediately notifies other third party services such as emergency medical service (EMS), caregivers, police station and fire station, if necessary. 

### 4.2. Interoperability and Standardization

**Figure 5:**
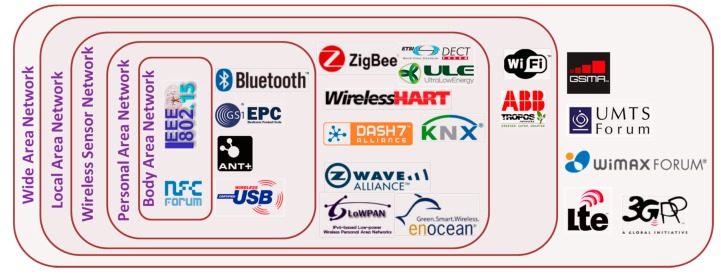
Fragmentation of wireless communication platforms.

One of the key concerns in adopting the IoT technologies for smart homes evolves from the fragmentation of the technologies [88,89,90]. The fragmentation of the IoT technologies, which is not only driven by technology constraints but marketing and business policies also [88,91] causes lack of interoperability among the smart devices, platforms and systems. These issues need to be addressed for ubiquitous adoption of the IoT in smart homes. Smart homes, as the term implies, are envisioned to be fully automated, energy efficient, and sustainable as well as capable of monitoring, assessing the health, safety and wellbeing of the occupants. It also requires a robust communication platform and might also facilitate assistance to the occupants for the ADLs. Therefore, the smart homes are expected to be equipped with a wide variety of devices, systems and platforms from different suppliers in order to provide the occupants with a wide range of services. However, the communication technologies used in those devices and systems may vary from supplier to supplier, thus leaving a fragmented IoT market and thereby posing a great challenge for the smart home service providers in bringing together different technologies in a cost-effective and energy-efficient manner. For example, there exists long-range cellular communication technologies such as GSM, EDGE, 3G, HSPA, and LTE along with several non-cellular short or medium range wireless connectivity solutions presented in Figure 5, while new technologies such as ABB-free@home^®^ [92] and Thread Protocol [93] are emerging. Each of these non-cellular technologies offer its own advantages and also has its limitations. However, the key concern evolves from the fact that they often are not compatible with each other. 

A common, extensible and standardized platform is thus required to ease the integration of different technologies, systems and services from different manufacturers. The internet of things, services and people (IoTSP) is such a platform that is particularly designed for building automation [88,94]. In fact, there exists a number of standards for the IoT developed by major standard development organizations (SDOs) such as Institute of Electrical and Electronics Engineers (IEEE), International Organization for Standardization/International Electrotechnical Commission (ISO/IEC), International Telegraph Union-Telecommunication Standardization Sector (ITU-T), Internet Engineering Task Force (IETF), and European Telecommunications Standards Institute (ETSI) [89,90]. Each SDO has their own point-of-view towards the IoT; however they are putting their efforts to bridge the gap among the standards. The interoperability issue is currently being addressed by adopting the internet protocol (IP) as the common platform, which, by assigning local IP addresses for the devices and systems, allows for realizing a cost-effective solution for device level connectivity and system integration [88,90]. BACnet/IP, KNXnet/IP, HomePlug, and Modbus TCP/IP (transmission control protocol /internet protocol) are some examples of IP-based wired communication technologies. There also exist some IP-based versions of wireless communication technologies such as IPv6 over Low-Power Wireless Personal Area Networks (6LoWPAN) over Bluetooth, ZigBee IP, 6LoWPAN over DECT ULE, and Thread. In fact, ETSI and the IPSO Alliance organized their fourth Constrained Application Protocol (CoAP) Plugtests™ event in London, UK in March 2014 [95]. They also organized the first 6LoWPAN Interop event in Berlin, Germany in July 2013 [96]. These events allowed the vendors to assess the level of interoperability of their systems and verified whether the IETF base specifications were interpreted correctly. The tests were performed using the 2006 release of the 2.4 GHz low-rate wireless personal area networks (LR-WPANs) PHY/MAC standards. Although all implementations were observed to send and interpret data correctly, they exhibited poor compliance with IETF RFC 6775, which describes optimization of neighbor discovery and addressing mechanisms for 6LoWPANs [97].

In addition, there is a growing consensus among the engineering and scientific community of using Representational State Transfer or RESTful web services to develop the application programming interfaces (APIs) for the IoT applications [88,89]. RESTful web services are light weight and highly flexible, which uses Hypertext Transfer Protocol (HTTP) for data communication. It allows the system to communicate with different devices in the network running on different communication platforms. It thus allows for building a bridging platform for all the sensors, actuators and systems used in the smart home, irrespective of the manufacturer and can successfully fulfill the integration requirements, which are critical for seamless operation of the smart home [98,99]. The adoption of RESTful web services in the IoT may also enable adopting other semantic technologies such as OPC UA (Open Platform Communications Unified Architecture) and oBIX (Open Building Information Xchange) from the internet industry in future. 

## 5. Smart Monitoring Systems for Elderly and People with Disability

**Figure 6:**
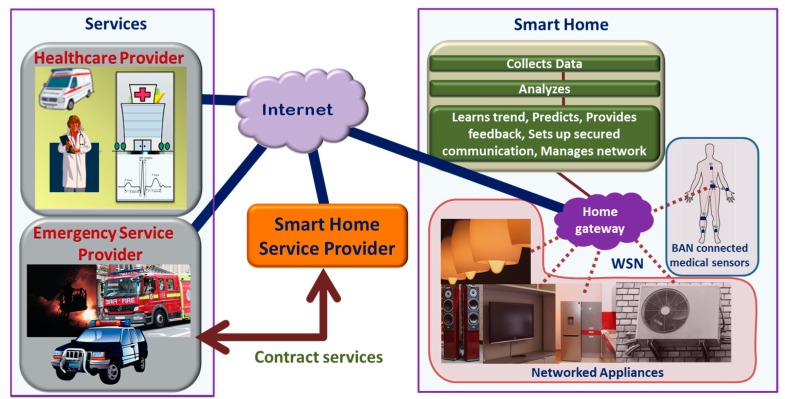
Schematic diagram of a smart home showing the network among different stakeholders.

As people age, often, their need for medical support grows, which may result in frequent and unplanned medical attention or in-clinic healthcare services. In order to get long term healthcare service, some elderly people need to stay in long term care (LTC) centers, which are expensive as well as of limited capacity. However, the ongoing development towards the IoT technology can play a pivotal role for the growth of elderly healthcare systems [100]. In a smart home, various key physiological signs of the elderly can be measured and monitored using simple, low-cost sensors from a remote healthcare service center over a secured communication platform, thus offering a cost-effective solution for long-term health monitoring. This will also allow the elderly to lead an independent life in their homes while ensuring maximum comfort, safety and security [19]. An illustration of a smart home solution used for elderly people is shown in Figure 6. 

The smart homes can benefit from artificial intelligence (AI), which can gather and analyze information regarding the occupant’s activities and health status, identify and report any anomalies. The AI system includes a database that stores residents’ behavioral and physiological patterns, and medical histories [101]. In case of a medical emergency, this system can raise an alarm and share medical profiles with the concerned authority over a secured channel, thus allowing the residents to have immediate and appropriate medical attention.

Recently, a wide range of wearable systems have been proposed for elderly healthcare that can monitor vital signs as well as the activities of the patients [17,18,52,102,103,104]. These systems include sensors software and wireless technology to collect, process, analyze and transfer physiological and activity related data to a remote healthcare center. Intelligent homes may incorporate BAN connected wearable systems as well as environmental sensors, actuators, and cameras, all connected through a WSN. 

### 5.1. Automated Emergency Call Systems

Some smart home solutions monitor the environment of the home as well as the physiological parameters of the elderly and can communicate with service providers in case of an emergency. The Allocation and Group Awareness Pervasive Environment (AGAPE) is such a healthcare system designed for patients living far from a healthcare facility [105]. When the AGAPE detects any anomalies in the data measured by the on-body sensors, it starts looking for and contact nearby caregiver groups. Once the group is informed, AGAPE locates the patient’s profile and forwards it to them. Meanwhile, AGAPE contacts and keeps other groups informed about the situation and requests additional assistance, if necessary.

A smartphone-based emergency calling system is presented in [106]. The system can generate an automated call to the EMS or caregivers in case of an emergency and provide them with the location information obtained from the embedded GPS. The emergency signal can be generated manually by the user or automatically by the system when a pre-defined threshold level is crossed, for example, room temperature exceeds or falls below a user-defined threshold. A combination of wireless devices and WSNs named INCAS (Incident-Aware System) is proposed in [107]. The system uses Raspberry Pi boards, motion sensors, cameras and a central server and can predict for any possible hazards in the house. The server collects data from sensors, analyzes and sends notification to the smartphone of the user in the form of sound, vibration or hazard images. Moreover, the system can be configured to call pre-defined numbers for assistance, if required.

### 5.2. Automated Activity and Fall Detection Systems

Smart homes need to distinguish between normal and abnormal activities with high accuracy in order to respond with appropriate actions. Some smart homes use video-based systems to monitor and recognize different activities [108,109,110,111]. Although, these systems can recognize complex gait activities, they restrict the user to reside within a specific area. In addition, these systems are expensive and require high processing resources [16,17]. Motion sensors such as accelerometers, gyroscopes and magnetometers, in contrast, are smaller, simple to use and low-power devices, thereby suitable for monitoring human activities in a wearable platform. These motion sensors along with vital signs sensors can be embedded in socks, armbands, t-shirts in order to receive comprehensive information about the overall health status of the subjects [102,111]. Other motion detectors such as passive infrared sensors (PIR) can be used to detect the location of the subjects in the house [112]. Rashidi et al. in [113] proposed a data mining technique for automatic activity recognition, which demonstrated high accuracy and sensitivity even in the presence of discontinuities and variations in the activity patterns. They used motion sensors as well as interaction tracking sensors to obtain quantitative information about activity patterns. The measured patterns were clustered into different activities which were later used to develop a Hidden Markov model (HMM)-based algorithm for activity recognition. A detailed review on motion sensor based activity detection systems can be found in [16,113].

**Figure 7:**
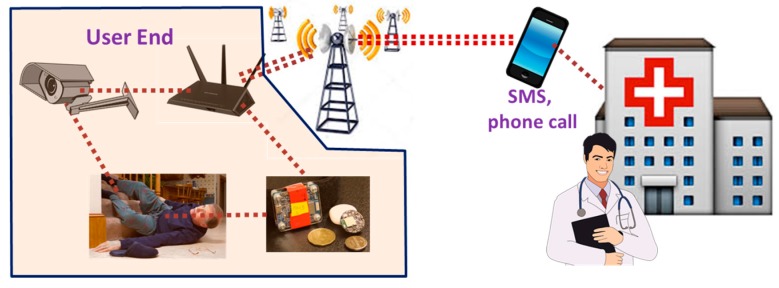
Remote fall detection system.

Falls are one of the leading causes of injuries and death among the elderly. In case of non-injurious falls, around 47% of persons experiencing a fall need external support to get up [114]. A general representation of fall detection system is shown in Figure 7. When the system detects a fall, it will inform the corresponding personnel by triggering an alarm. An acoustic fall detection system (FADE) is presented in [115], which detects a fall based on the sound of a fall. The system uses several acoustic sensors mounted vertically to detect fall and a motion detector to enhance detection accuracy. A fall detection system based on wearable sensors is proposed in [116], which works through consumer home networks. The base system relies on a micro-programmed controller unit (MCU), which detects a fall based on the measurement obtained from accelerometers and other environmental sensors.

In [117], a smartphone based fall detection system, which uses the accelerometer embedded in the smartphone to measure movement of the user and analyzes the data to detect a fall was developed. Once a fall is detected, the system notifies the authorized personnel. A three step fall detection system using multimodal signal sources was presented in [118]. The system uses on-body accelerometers for primary detection of fall and later verifies it by activating a microphone and camera to capture voice and images of the subject, respectively. The system makes a decision based on the multimodal information and sends an e-mail to a doctor and relatives, as required.

### 5.3. Vital Signs Monitoring Systems

**Figure 8:**
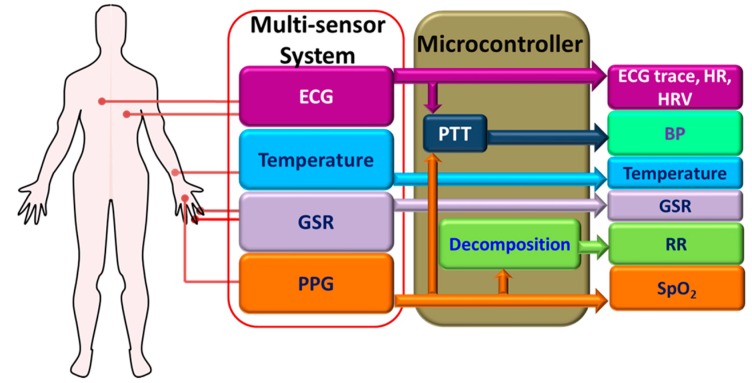
Four sensor health monitoring system [16].

Vital signs, which include heart rate (HR), body temperature (BT), respiration rate (RR) and blood pressure (BP), are the most basic parameters that are routinely monitored by the medical professionals to get a good overview about the health of the patients. Several vital signs monitoring systems are reported in the literature. However, most of the systems are designed to measure and monitor one or two specific parameters only. For example, only ECG and HR were measured and monitored in [36,119,120], and only body temperature is monitored in [121,122]. Using separate systems for each parameter is impractical and may cause inconvenience to the user, particularly for continuous and long-term monitoring. A concept of multi-parameter monitoring system in a wearable platform is proposed in [17]. The authors envisioned that a detailed set of physiological parameters such as ECG, HR, HR variability (HRV), BT, BP, GSR, RR, and SpO_2_ can be measured and monitored in real-time by using only four sensors: ECG, PPG, GSR and BT sensor (Figure 8).

Smart beds embedded with vital signs sensors are other attractive solutions for monitoring elderly health as well as their sleep quality during sleep [123,124]. An un-obtrusive sleep monitoring system was proposed in [125] that employed a grid of pressure sensors underneath the bed to detect body movement and sleep patterns. They exploited machine learning techniques to detect different sleep stages and patient’s position on bed. A similar system based on a tri-axial accelerometer and a pressure sensor was presented in [126]. In addition to detecting different sleep stages, this system can estimate the depth of sleep, number of apneic episodes and periodicity, and detect early symptoms of sleep disorders. A detailed review on smart beds based on piezoelectric and pressure sensors can be found in [127]. Although these systems are useful to estimate the quality of sleep and some of them used the pressure sensor data to estimate the RR [126], they are not capable of providing detailed information about the vital signs.

A non-contact proximity vital signs sensor for measuring HR and RR was proposed in [128]. A circular resonator was used in the monitoring system as the antenna, which also worked as a series feedback element for the voltage controlled oscillator (VCO) that controls a phase locked loop (PLL). The distance between the antenna and the body varies with the movement of the chest during respiration and heart activity, thus changing the input impedance of the resonator. The oscillator frequency thereby changes accordingly with the variation of the resonator input impedance. The authors were able to measure RR and HR at a distance of 50 mm from the dorsal side, which makes it a potential candidate for embedding in beds, chairs or garments for non-contact, un-obtrusive HR and RR monitoring. Another non-contact vital signs monitoring system was proposed in [129] that used a wireless signal and its variation in reflection time from the body to estimate the chest movement, and thus RR and HR. The authors reported to achieve an estimation accuracy of 90% at a distance from 8 m. A detailed review on wearable vital signs monitoring systems was presented in [16].

### 5.4. Reminding Systems

Memory and cognitive function in the older adults decline gradually with age [130] causing many elderly people to suffer from severe memory loss and dementia. This loss of cognitive functionality can disrupt their daily living and even be dangerous at times, for example, if a person forgets to take the medicine or takes higher doses than prescribed. Therefore, a reminding system would be very useful for the elderly in their daily life. The system raises an alert signal at a pre-scheduled time and can send detailed information to the user or the caregivers, as needed [131].

Wedjat is such an application that is designed to remind the individuals about their medicines as well as meals [132]. The application takes the prescription as the input and reminds the patient to take the medicine about 1 to 15 min before the scheduled time, provides in-take directions and keeps records of all taken and missed medicines. An activity tracking application in android platform for smart homes was presented in [133]. The system has a reminder application for the elderly and a separate application for the caregivers or the family members of the elderly. The application reminds the elderly about medicines, and scheduled tasks. It also notifies the caregivers or family members for assistance in case of critical situations. 

A hardware based medication reminder system is proposed in [134]. This system reminds the patient about the medicine at a prescheduled time, provides them with appropriate dose of medicine, and gives vocal guidance about the in-take procedure. The system uses sensors and actuators to monitor the patient’s activities and control the medicine dispensing units with right amount of dose. It also can facilitate communication with the caregivers if necessary. 

### 5.5. Automated Health Assessment

The automatic and continuous assessment of the cognitive and physical health of the residents is one of the key services that the smart monitoring systems can facilitate. Continuous monitoring and real time assessment of health can be useful in balance and fall analysis, rehabilitation following an injury, and can also enable early detection of physical and cognitive impairment. For example, gait patterns tend to differ from its normal behavior at the early onset of some neurodegenerative diseases, such as Alzheimer’s and Parkinson’s [135,136]. A person at the primary phase of Parkinson’s tends to make small and shuffled steps, and may also experience difficulties in performing key walking events, such as starting, stopping, and turning [16,17]. Therefore, quantitative assessment of daily activities, gait patterns, and vital signs can be very useful for early detection of a potential health problem. 

The smart monitoring systems can integrate automated activity monitoring and vital sign monitoring systems to evaluate the overall health status of the residents with the help of modern machine learning techniques. The automated assessment algorithm generally begins with the extraction of key parameters/features from the sensor data associated with a particular activity or physical health. These features are then used by an appropriate machine learning algorithm to make quantitative assessment about the overall health status. For example, researchers in [137,138] exploited empirical mode decomposition (EMD), and Complete Ensemble EMD (CEEMD), respectively to decompose and extract features from the walking signals acquired by on-body accelerometers and gyroscopes. The high dimensionality of the feature set was reduced to a two-dimensional vector by employing principal component analysis (PCA) on the features. The system then employ KNN algorithm on the first two principal components to analyze, cluster and assess human gait based on their age. 

An automatic assessment algorithm was presented in [139] for evaluating human cognitive health based on the activity information measured by infrared detectors and magnetic door sensors. The researchers extracted several features corresponding to some specific daily tasks and employed both supervised and unsupervised learning approaches on the extracted set of features to classify the test subjects into three groups based on the level of their cognitive health. Rabbi et al. presented an automated system for evaluating mental and physical health by quantifying behavioral features measured by a mobile audio sensor in a normal everyday setting [140]. The researchers calculated the total amount of human speech in the audio recording, evaluated the subjects’ mental health using a traditional paper-based survey and performed a comparative analysis using univariate regression technique. The total amount of speech was observed to be highly correlated with the subjects’ mental health and therefore, can be used as a potential indicator of mental health.

## 6. Smart Homes for Elderly Healthcare: Prototypes and Commercial Solutions

**Figure 9:**
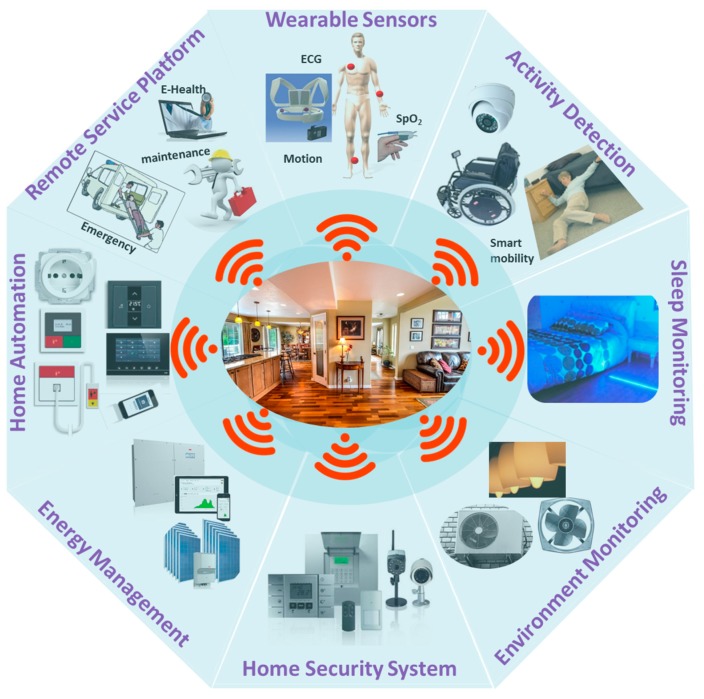
Smart homes integrated with automated systems for elderly healthcare.

In the above discussion, we have presented different healthcare and monitoring systems reported in the literature. As depicted in Figure 9, a fully-fledged smart home requires all such systems along with a wide range of physiological and environmental sensors to be integrated in a common platform that poses new challenges in terms of volume of information, uninterrupted connectivity, interoperability, and most importantly, privacy and data security [19,141,142,143]. Many researchers along with some technology companies around the world have been working to overcome these technological challenges. In this section, we present some prototypes of smart homes reported recently in the literature. We also discuss some commercial smart-home solutions currently available in the market.

### 6.1. Smart Home Solutions in the Literature

The University of Colorado, Boulder in one of the earliest smart home projects, explored the concept of a self-automated home [144]. The researchers developed a prototype that is capable of monitoring and controlling the temperature, water, ventilation system and lighting in the home. The researchers exploited neural networks to learn and predict the behavioral patterns from the lifestyle of the residents and to accommodate the needs of the occupants accordingly. A smart home system, MavHome (Managing An intelligent Versatile Home) was introduced in [145] that used several sensors to perceive and analyze home environment as well as the residents’ action. The MavHome intelligent agent learns the patterns observed in the residents’ activities and developed a statistical model to make predications and control the home environment accordingly. 

Researchers at University of Florida, Gainesville developed a programmable pervasive computing space for the smart home (GatorTech Smart House) that allows automatic integration of system components [146]. They developed an extensible and easy-to-integrate service framework, which contains service definitions for all system components, thus enabling automatic discovery and integration of the components. The system uses a multi-layered architecture to discover and communicate with the sensors and actuators. The architecture also facilitates analysis of sensory data and contextual information to provide an intelligent assistive living environment for the occupants. One may find other early implementations of smart homes in [73,147], which includes the ‘Intelligent Workplace’ at Carnegie Mellon University [148], Georgia Tech Aware Home [149], Smart Medical Home at University of Rochester [150], and MIT intelligent room project [151].

Researchers from Carleton University, Ottawa presented an overview of smart home system for monitoring the elderly health and wellbeing [152]. They developed an initial prototype of smart home by installing and integrating several sensors such as magnetic switches, infrared motion sensors and pressure sensitive mats to monitor the home environment and security. They proposed a four-tier alarm system based-on the severity of the detected anomalies. Another group of researchers from the same and other universities studied the feasibility of integrating the IoT with web-based services and cloud computing [152,153]. They installed actuators to control lights and fans as well as several sensors such as temperature, humidity, ambient light and proximity sensors to monitor home environment. All sensors and actuators were connected over the ZigBee protocol and can be monitored or controlled over a cloud-based computing service.

The design and implementation of a mobile healthcare system (mHealth), particularly for wheelchair users was presented in [154]. Several environmental sensors, actuators for appliance control and cameras were installed in a 6 m × 6 m room. The user wore a HR sensor as well as an ECG sensor, which facilitate cardiovascular activity measurement. A wireless pressure cushion and accelerometer were installed in the wheelchair to detect the falls and for activity monitoring. The researchers also developed an Android-based software interface to monitor and display the physiological signs as well as to control the home environment by activating the actuators. The software collaborates with a third-party service to send text messages and voice calls in case of an emergency. 

An advanced platform for in-house health monitoring and assessment called The ORCATECH Life Laboratory was developed by the Oregon Center for Aging and Technology (ORCATECH) [155]. The researchers mostly exploited commercial ambient and passive wireless sensors to monitor and assess the physical and cognitive health of the occupants. The sensors were connected to a wall-mounted hub, which functioned as the gateway of the smart home and was able to transmit the measured data to a cloud-based server over the internet or 3G mobile communication protocols. The computing and decision making layer was implemented in the server, which processed and analyzed the sensor data and ran advanced machine learning algorithms to assess occupants’ overall health status based on several parameters such as walking speed, sleep quality, and activity.

A WSN-based smart home, designed for elderly health care was proposed in [156]. The smart home comprises a set of wireless sensors, which facilitates monitoring the temperature and safety of the home. The system used ZigBee technology for implementing the WSN and was capable of raising an alarm in case of an emergency. A smart home platform was developed in [157] primarily for monitoring home environment and residents’ activities. The system deployed cameras and wearable inertial measurement unit (IMU) to measure and assess movement patterns. All sensors were connected over the Bluetooth Low Energy (BLE) and IEEE 80 2.15.4 platform, whereas the gateway, video camera and computers were connected over the WiFi. The gateway supports both IEEE 80 2.15.4 and WiFi and functions as the hub for all sensors, and computers in the home. It also can communicate with a remote data hub through a virtual private network (VPN). 

A prototype of smart home, presented in [158], was capable of monitoring several physiological signs such as ECG, BP, SpO_2_, and BT along with few environmental parameters and appliances. The sensors transmit measured data over the BLE to the gateway, which then communicate with the upper layer for storage and further processing. The system also includes a clinical governance system that brings both the clinicians and patients in a common platform. It reminds the patient about a scheduled measurement set by the clinicians, notifies the clinicians about any anomalies in the measured data. Table 3 presents a comparison among several smart home systems reported in the literature in recent years.

### 6.2. Commercial Solutions for Remote Elderly Care

Several remote elderly care solutions are currently available in the market. GreatCall Responder is a small, GPS-enabled device that can easily be attached to a keychain, purse or backpack [159]. It provides an easy and convenient way to safeguard an elderly at home and on-the-go. The user can communicate with a trained service agent by pressing a button on the responder. The agent then assesses the situation and takes further necessary actions. The system also allows the user to contact the EMS directly. MobileHelp [160] uses a GPS-enabled wearable system and offers similar services as GreatCall offers. GrandCare provides in-home healthcare and caregiver services for their clients [161]. The system communicates with the wireless sensors installed in the residence over the internet. Caregivers can log into the GrandCare website to check the health status of the residents. The caregivers are notified if any unusual activities are detected. They also offer a wide range of services including communication and entertainment services to their clients. BeClose remote monitoring system, which is currently owned by Alarm.com [162], is designed to keep elderly in close contact with their family and caregivers [163]. The system uses discrete wireless sensors placed at different locations in the home to track the daily activities of the elderly. The caregivers or family members of the elderly can also monitor his/her activities using a private and secure webpage. The system can notify the caregivers by phone calls, e-mails or text messages in case of any emergency.

CareSmart Seniors Consulting Inc. (Kelowna, BC, Canada) offers remote monitoring services for the elderly [164] by using a wireless monitoring system from Care Link Advantage [165]. The system utilizes cameras to track the activities of the elderly residing at home or to determine the level of urgency. They consult with the elderly and his/her family to identify the areas of concern and program the system for generating notifications accordingly. In the case of an issue, notifications are sent to the family members and the caregivers via e-mails, text messages and voice messages. Independa offers cloud-based elderly care services through a software platform that uses a smart TV to connect the elderly with the caregivers or the family members [166]. The resident uses a traditional remote controller to switch between TV shows and one of the Independa services. It offers communication services such as video chat, photo sharing, message and alert call between the elderly and the family members. It also reminds key events such as important activities of daily living (ADL), appointments with doctors, social engagements, and schedule of medication.

Currently, many leading communications and media companies such as Rogers Communications [167], Bell Canada [168], AT&T [169] and British Telecom (BT) [170] are offering smart home solutions to their customers. Although these solutions offer excellent services for monitoring the safety and security and controlling the environment and the appliances of the home, they still lack comprehensive healthcare monitoring services. Some technology companies such as Philips [171], ABB [172] and Iqarus [173] are offering remote healthcare services and medical solutions. However, these solutions are primarily designed for large-scale clinical environments.

Samsung, one of the pioneer technology companies, has been working to create a unified platform for elderly care solutions. The platform is designed to be interoperable between Samsung and other devices [174]. With the aid of this unified platform, they are expecting to provide personalized, simple and easy-to-use healthcare solutions, thus offering better care, independence and improved life style for the seniors. Along with ensuring regular communication between the elderly, family members and the healthcare staffs they will also offer seamless connectivity between SMART TVs and appliances, medical alert services, measurement and monitoring of home environment, physiological signs and activities of the seniors.

## 7. Research Challenges for Smart Homes

Smart homes allow continuous monitoring of health and activities of the elderly at home as well as monitoring of the environment, safety and security of the home. Although researchers have been working towards a fully functional smart home, there are some challenges that need further research and development in order to improve the overall performance and increase the market penetration of the smart home systems.

First, one of the most pressing concerns for the smart home technologies is associated with the privacy and security of the transmitted data. The data may contain sensitive, protected or confidential information that can endanger residents’ privacy and safety, if breached. Therefore, ensuring strong data encryption, database security as well as secured communication channels is critical for smart homes.

Second, smart homes use a wide range of sensors, actuators and other wireless devices, thus generating a large volume of data. Therefore, the communication protocols, hardware and computation resources for the central node of the body area network and wireless sensor network could impose bottlenecks for the seamless and delay-less connectivity as well as data handling capability. The gateway node in wireless sensor network performs extensive data processing as well as communicates with all the components of the system along with the remote server. Robust and efficient algorithms along with effective data compression techniques are the key to optimize the performance of the smart home system.

Third, smart home is a complex system with many discrete devices and systems connected in a common platform. However, the system needs to be carefully designed to deal with integration issues among different devices and also to have optimum number of sensors in order to avoid redundant data, minimize infrastructure and maintenance cost as well as energy consumption without losing key information.

Fourth, the sensing systems of the smart homes, particularly the portable and wearable physiological parameter measurement systems, are aimed for long-term monitoring purposes. Therefore, these systems need to be energy efficient, which can be achieved by using low-power components and efficient batteries. Researchers may also exploit energy harvesting techniques to fulfill the energy requirements of the devices. 

Fifth, modularity, expansion capability of the system and interoperability among different smart home platforms are vital for achieving flexibility and widespread acceptance among the users. A modular and extensible structure will allow the users to choose the components from different manufacturers or add/remove services. A common or inter-operable platform for all types of sensors and systems in smart homes is necessary to achieve modularity as well as to ensure flawless and seamless operation. Although, there exists several hardware-based and IT-based standards at present, they must be converged towards a global common standard to unfold the full potential of the IoT in smart home and to lay a level playing field for the business competitors as well as the customers. 

Sixth, the adoption of AI technologies in the computational platform of the smart home would potentially play a pivotal role in realizing a fully automated and self-sustainable solution. AI technologies, through continuous learning and assessment of the occupants’ physiological and behavioral patterns as well as the home environment, will allow the smart homes to make prediction, recommendation and decision about the health, safety and security of the occupants. However, ensuring a highly reliable, accurate and robust implementation of AI technologies particularly for decision making and execution purposes is critical for a trustworthy and safe operation of the smart homes. In addition, in order to make the best use of AI driven features such as machine learning, robotics and big-data computing in the smart home, standardized protocols need to be developed and implemented.

Finally, although many researchers have been working towards smart homes, they mostly addressed some specific aspects of smart homes. A fully functional and comprehensive smart home that addresses all aspects such as home automation, monitoring of residents’ health, safety and security, and home environment is still to be realized. 

## 8. Future Perspectives and Conclusions

In this paper, we have presented a review on the state-of-the-art technologies for elderly care in smart home platforms. The primary objective of the smart homes is to allow the elderly to receive continuous, non-invasive and seamless healthcare service while staying in their convenient home environment. It allows the elderly to minimize their frequency of visits to, or length of stay in expensive healthcare centers such as clinics, hospital and long term care centers, thereby allowing them to lead independent and active lives. Smart homes can also monitor and control the home environment by assessing the behavioral and daily living patterns of the users. The significant advancement in the technology that enables the development of low-power, small and low-cost sensors, and actuators coupled with modern communication technologies paved the way towards realizing continuous monitoring services in a smart home platform from a distant facility. 

Smart homes can provide comprehensive information about the overall health status of the elderly through continuous monitoring. Modern low-cost sensors, actuators, computing and communication technologies are the key for developing fully functional smart homes. The system may also include predictive algorithms in future, which will allow it to make predictive decisions about diseases at their early onset by analyzing the monitored data. If a potential health problem is predicted, the system can notify the corresponding healthcare personnel immediately over a secured communication channel for a detailed investigation. This may enable the individual to receive early diagnosis and prevent treatment delay. Researchers may exploit data fusion techniques, which integrate data/information from different sources to develop a predictive tool with a high degree of prediction confidence. The data/information fusion techniques may also allow the system for context-based learning of the residents’ daily living and health trends in the smart home.

A key concern for the seamless operation of the smart home system is associated with its energy requirement. Low power consumption and high energy efficiency are critical for the smart home, especially for the wearable and mobile systems used for long-term monitoring purposes. Advanced battery technologies as well as low-power electronic components can be used to increase the operating-time of the system. Researchers also may put their efforts into developing and integrating efficient energy harvesting technologies to fulfill the energy requirements of wearable and mobile systems in the smart home. 

Most of the standalone products which are currently available in the market are proprietary and generally developed for one or a few specific tasks or functionalities. Although these systems use standard communication protocols, they are mostly not compatible to, or interoperable with similar systems from other manufacturers, thus leaving the consumers with few alternatives. A common platform for all systems will raise the competition among the manufactures that will result in many alternatives for the consumers, thus increasing the market penetration of smart homes. Therefore, a global industry standard based on a well-defined layered architecture is critical for the widespread acceptance of the smart home technology. Researchers and industry groups may work together to develop and adopt a common and unified industry standard for the smart home system.

Furthermore, as the smart textile technologies continue to evolve, wearable healthcare systems based-on smart textiles are expected to be an attractive solution for comfortable and un-obtrusive monitoring of health parameters in a smart home platform. Textile-based sensors or smart textiles can be fabricated using conventional textile technologies such as weaving, printing, knitting, and stitching, thus having a great potential for developing low-cost wearable sensors. However, further research and development is required to improve the sensitivity, durability, stability, signal-to-noise ratio and reproducibility of the textile-based sensors for using them in long-term monitoring systems. 

In recent times, with the development of high performance miniaturized sensors, actuators, computing processors there is a growing interest in implementing innovative and futuristic technologies such as robotics, artificial intelligence (AI) and 3D printing in the healthcare sector. A caring robot driven by AI can assist the elderly in their daily living without any intervention from a third-party system and potentially be a very useful addition to the smart home. 

Big communication and media companies, who already have high market penetration and robust infrastructures for high speed and secured data communication, may collaborate with third-party healthcare service providers such as hospitals, clinics, and ambulance services to bring healthcare facilities to the doorsteps of the people. An addition of comprehensive health monitoring systems and healthcare services to their existing smart home solutions can potentially be a giant leap towards a ubiquitous and fully-functional smart home. In fact, some major technology companies such as Samsung, Alarm, and ADT (founded as American District Telegraph) have acquired several small smart home companies in recent years to facilitate health monitoring along with their existing home-security applications in the smart home platform. Also, the industry is still actively working to realize a fully functional smart home-based remote healthcare solution.

Finally, manufacturers also need to pay attention to the design aesthetics in addition to the performance and ease-of-use of the installed devices and systems. A home reflects an individual’s personal identity and also creates a sphere of physical and mental comfort for the occupants. Therefore, a superior system with poor visual aesthetics may not be well accepted by the consumers. The architects may also make use of the false walls, interior ceiling, and false ceiling while designing the interior of the home to hide and protect the installed devices and systems, thus providing the occupants with a sense of visual comfort.

Overall, a smart home is a complete system that is expected to bring healthcare, safety and well-being services to the user’s doorstep with the aid of modern technologies such as environmental and medical sensors, actuators, high performance computing processors, and wireless commination platforms. The system exploits the concept of Internet-of-Things and connects all sensors and systems of the home to facilitate remote surveillance of the occupants’ health as well as the environment, safety and security of the home. Although several standalone systems such as vital sign monitoring, emergency call and reminding systems are available, a fully-fledged smart home is still far from the reality. Therefore, more research and development is required in this sector to develop a fully-functional smart home while ensuring system reliability, privacy and data security, robustness of processing and prediction algorithms, seamless connectivity with minimal transmission delay, energy-efficiency and low setup and maintenance cost. 

## Figures and Tables

**Table 1:** Some examples of WBAN applications in the literature.

**WBAN Applications**	Wearable WBAN	Monitoring activities of soldiers in the battlefield by WBAN by using sensors, cameras and wireless technologies [31]. Monitoring harsh environments by policemen and fire-fighters in order to reduce the casualties [32]. Real-time health monitoring. For instance, the cell phone of a diabetic patient can detect the glucose and send it to a doctor for analysis [33].
	Implantable WBAN	Myocardial Infarction (MI) can be reduced by monitoring episodic events and other abnormal conditions through WBAN technologies [34].
	Remote Health Monitoring	WBAN can be connected with a medical care facility over the internet in order to monitor health conditions, thus reducing the dependency of patients on in-clinic monitoring [35]. Integrating WBANs in a telemedicine systems to promote ambulatory health monitoring [36].

**Table 2:** Communication technologies for smart homes.

Wireless Tech.	Frequency	Range	Data Rate	Power (mW)	Maximum Nodes	Network Topologies	Security
**RFID**	13.56 MHz 860–960 MHz	0–3 m	640 kbps	200	1 at a time	peer-to-peer (P2P) passive	N/A
**Bluetooth**	2.4–2.5 GHz	1–100 m	1–3 Mbps	2.5–100	1 M + 7 S	P2P, star	56–128 bit key
**BLE**	2.4–2.5 GHz	1–100 m	1 Mbps	10	1 M + 7 S	P2P, star	128-bit AES
**HomePlug GP**	1.8–30 MHz	~100 m	4–10 Mbps	500	-	P2P, star, tree and mesh	128-bit AES
**EnOcean**	902, 928, 868 MHz	30–300 m	125 kbps	~0.05 with energy harvesting	-	P2P, star, tree and mesh	128-bit AES
**ZigBee**	2.4–2.5 GHz	10–100 m	250 kbps	50	65,533	P2P, star, tree and mesh	128-bit AES
**WiFi**	2.4–2.5 GHz	150–200 m	54 Mbps	1000	255	P2P, star	WEP,WPA, WPA2
**DASH7**	315–915 MHz	200 m–2 km	167 kbps	<1	-	P2P, star, tree and mesh	128-bit AES
**Insteon**	RF: 869.85, 915, 921 MHz powerline: 131.65 KHz	40–50 m	38 kbps (RF) 2–13 kbps (powerline)	-	64,000 nodes per network	P2P, star, tree and mesh	256-bit AES
**Sigfox**	868/902 MHz	10–50 km	10–1000 bps	0.01–100	-	P2P, star	No default encryption
**NFC**	13.56 MHz	5 cm	424 kbps	15	1 at a time	P2P	AES
**Wireless HART™**	2.4 GHz	50–100 m		10	-	P2P, star, tree and mesh	128-bit AES
**6LoWPAN**	2.4 GHz	25–50 m	250 kbps	2.23	-	P2P, star, tree and mesh	128-bit AES
**ANT**	2.4–2.5 GHz	30 m	20–60 kbps	0.01–1	65,533 in one channel	P2P, star, tree and mesh	64-bit key
**Z-Wave**	860–960 MHz	100 m	9.6–100 kbps	100	232	mesh	128-bit AES

AES: Advanced Encryption Standard.

**Table 3:** Smart home systems.

Ref.	Proposition	Country (Year)	Resident activity Monitoring	Home Environment Monitoring	Resident Health Monitoring	Home Appliance Monitoring	Wireless Connectivity	Summary	Alert/Reminder Service
[116]	Fall detection system for smart home	China and Korea (2014)	- Accelerometer: activity and fall detection	Temperature and humidity sensors	Pulse pressure sensor: HR		ZigBee with multiple access points		Proposed but not implemented
[133]	Daily activity tracking for smart home	Korea (2012)	RFID Tags and self-developed biosensor and logging system				RFID	Developed applications in android platform for tracking ADL of the elderly	Smartphone based application for the elderly and the caregivers, family
[153]	Smart home based on cloud computing	Canada (2013)	- Proximity sensor	Temperature, humidity, ambient light		Light and fans	ZigBee, RFID	Arduino-based application communicates with the user, sensors, and actuators as well as interacts with the cloud-based computing service	
[154]	Mobile healthcare system for wheelchair users	China and Canada (2014)	- Pressure cushion: fall detection Accelerometer: embedded in wheelchair to detect the falling of wheelchair IR sensor: location detection Camera: activity detection	Temperature, humidity, smoke sensor	- ECG sensor module Photoelectric pulse sensor: pulse measurement	Lights and air conditions	ZigBee and Bluetooth	User can interact with the home environment remotely and locally via smart phones	Connected to a third-party service to notify emergency situation using SMS and telephone
[155]	Cloud-based platform for assessing elderly health and wellbeing	USA (2004 to date)	- PIR sensor: mobility and sleep monitoring Medication tracking computer and telephone usage tracking	Air quality and room temperature	Weight, heart rate, and body mass index		Bluetooth, WiFi, Zigbee	Developed a cloud-based cognitive and physical health assessment platform using mostly commercial ambient and passive sensing technologies.	
[156]	Smart home for elderly care	India (2015)	- Temperature sensor: fire detection, Gas sensor: gas leakage detection, Contact sensor: door monitoring				ZigBee	Developed an Arduino based software	- Warning message generates, and played through a loudspeaker SMS sent to the caregiver over the cellular network
[157]	Sensor platform for healthcare services in a home environment	Bristol, UK (2016)	- Vision sensors: track people and provide information Wearable IMUs: measure movement patterns and quality of movement	Temperature, humidity, luminosity, noise level, air quality, occupancy		Electricity metering, cold and hot water consumption	BLE , IEEE 80 2.15.4, WiFi	- BLE and IEEE 80 2.15.4 : for sensors and a 5 GHz WiFi : communications among the Home Gateway, video NUCs and tablet Prototype installed in home. Control and monitored parameters are sent to the remote system over VPN link	Remote system generates the alerts
[158]	Home tele-monitoring of vital parameters and detection of anomalies in daily activities	Milano, Italy (2017)	- Commercial solution for fall detection		ECG , BP and SpO_2_ weight, ear temperature, glycaemia	Water tap, refrigerator, and dishwasher	Bluetooth LE	Developed a clinical governance system to interact between patient and clinicians	Clinical governance system generates and displays alerts both to the patient and the clinicians
[175]	Cloud-based home healthcare	USA and China (2016)	- Smart watch, IMU: body activity recognition Acoustic sensor: hydration monitoring, PIR sensor: location detection		ECG, SpO_2_		ZigBee	Cloud based service for storage, processing and interaction with healthcare personnel	
[176]	Activity and physiological parameter monitoring and social interaction	Sweden, Italy, Spain (2014)	- PIR sensor: detects location Electrical usage sensor Pressure pads: occupancy detection Fall detection		- Android-based system for weight, blood pressure, glycaemia, pulse rate and SpO_2_	- Door contact sensor	Not mentioned	A telepresence robot with camera, lens, microphone and LCD screen to facilitate video communication with caregivers	Context recognition for event detection, trend analysis and alert generation
[177]	Monitoring and interactive robotic system	Spain (2015)	- Depth sensor: 3D position estimation of the occupants for detecting falls, abnormal behavioral pattern, intrusions					- A GUI is developed to transmit alarm message to relatives, caregivers or medical staffs	Alarms are programmed for fall, intrusion and abnormal pattern detection
[178]	Behavior and wellness prediction	New Zealand (2013)	- Force sensor: monitor bed, chair, toilet and sofa			Microwave, water kettle, toaster, room heater, TV	ZigBee	Software for data acquisition, activity recognition, behavior recognition and wellness determination are developed in C#	
[179]	Easy-to-install and lightweight smart home kit	USA (2013)	Infrared motion/light sensor, relays, Door sensor and temperature sensor				ZigBee	Developed a activity visualizer software to display and keep record of the ADL	
[180]	In-home Health Monitoring System	Japan (2015)	IR motion sensor, water flow sensor for monitoring urination, kitchen work, and washing				RFID	Monitors and assesses occupant’s health status by monitoring urination, kitchen work, washing activities and movements in the house	Generates and e-mail report on the occupants’ health condition
[181]	Health monitoring using data fusion techniques	France (2011)	- Microphones: acoustical monitoring of the elderly Wearable device: detect posture (standing/sitting and laying), fall and activity rate Infrared sensors: location, posture and movement detection	Temperature sensors	Wearable device: HR		ZigBee	- Software developed in LabWindows/CVI using multi-sensor data fusion technique Communicates with the wearable device and Gardien sub-systems: used TCP/IP and appropriate application protocols. Gardien: implemented in C++, recovers data every 500 ms.	
								Data fusion based on fuzzy logic: detect several distress situations	
